# Microwave-Assisted Rapid Synthesis of Metallic Iron Nanoparticles from Triiron Dodecacarbonyl

**DOI:** 10.3390/nano16060353

**Published:** 2026-03-13

**Authors:** Ehsan Ezzatpour Ghadim, Yisong Han, Festus Mathuen Slade

**Affiliations:** 1 School of Engineering, University of Warwick, Coventry CV4 7AL, UK; 2 Department of Physics, University of Warwick, Coventry CV4 7AL, UK; yisong.han@warwick.ac.uk; 3 Department of Chemistry, University of Warwick, Coventry CV4 7AL, UK; 4 Cambridge Neuroscience, University of Cambridge, Cambridge CB2 1TN, UK

**Keywords:** zero-valent iron nanoparticles, rapid synthesis, reaction control, microwave reactor, [Fe_3_(CO)_12_]

## Abstract

Zero-valent iron (Fe(0)) nanoparticles have a wide range of applications, including catalysis, energy storage, and even reported roles in human neurochemistry. This study demonstrated that [Fe_3_(CO)_12_] dissolves in N,N-Dimethylformamide (DMF) within a minute to resolve the dissolution problem of this complex. Dodecylamine (DDA) was used to produce DDA-coated Fe(0) at 383 K in 30 s with a microwave reactor. The powder X-ray diffraction (PXRD) of the Fe(0) profile indicated a pure-phase face-centred cubic (FCC) structure with *Fm*$\bar{3}$*m* space group. Varying the synthesis time from 30 s to 5 min did not significantly affect the unit cell parameters (3.5276 (±0.0001) and 3.5391 (±0.0001) Å). Microwave use yielded well-dispersed, pure Fe(0) nanoparticles, and the particle size, shape, elemental analysis, and surface oxidation of the Fe(0) nanoparticles were studied using scanning electron microscopy and dispersive X-ray spectroscopy (SEM/EDX). Annular Dark-Field Scanning Transmission Electron Microscopy (ADF-STEM) and Fourier-transform infrared (FT-IR) spectroscopy confirmed the surface coating of Fe(0) nanoparticles with DDA. Thermogravimetric analysis (TGA) was used to demonstrate the surface adsorption of DDA on Fe(0) nanoparticles. In addition, STEM showed that the average nanoparticle size under the stated synthesis conditions was 25.7 nm. This comparatively straightforward procedure offers advantages over existing practical approaches to the synthesis of Fe(0) nanoparticles, including safety, speed and reaction control.

## 1. Introduction

Metallic iron particles have garnered widespread interest due to their diverse applications, including environmental remediation, magnetic devices, and catalysis [1,2,3]. In addition, they have been detected and characterised in biological systems, even in the human brain, although their origin (biogenesis or environmental) and function remain unclear [4]. However, pure iron particles rapidly oxidise and aggregate upon exposure to air, which affects their magnetic and reactive properties [5]. Efforts have been made for synthesise metallic iron particles that mitigate these effects, including through nitridation, surface modification, and dispersing Fe(0) in stabilising matrices [6,7,8].

Approaches to synthesising Fe(0) include both conventional and unconventional methods. Top-down methods, such as mechanical milling, generate nanoparticles from bulk iron(0); however, the production faces extensive aggregation of particles [9]. Nevertheless, there are some barriers to the use of iron precursors, including [Fe(CO)_5_], due to the dissolution process, safety, and sensitivity to light and oxygen [10]. [Fe_3_(CO)_12_] is preferentially used due to its low volatility, and this iron–carbonyl cluster is more stable, easier to handle, and less toxic than [Fe(CO)_5_] [11]. Based on the reducing agent, the reduction of Fe(II) and Fe(III) compounds with NaBH_4_ can readily generate Fe(0) particles; however, it is a toxic process and expensive for large-scale synthesis. Alternative methods for developing safer, more cost-effective reductant agents and stabilisers are being actively explored [12].

Unconventional processes include chemical vapour deposition (CVD) at elevated temperatures, ultrasound-assisted reduction, and laser ablation in ionic liquids; however, they do not appear to offer any advantages over the more conventional processes for the rapid production of pure-phase iron [13,14,15].

Current materials chemistry prioritises low-energy, resource-efficient syntheses that minimise thermal budgets and processing time, making controlled-heating routes attractive for rapidly forming metallic nanoparticles under mild conditions [16,17]. Microwave radiation was discovered accidentally in sonar and radar systems and rapidly developed to personal use for cooking and for use in academic laboratories to synthesise chemical compounds [18,19]. Transforming the electromagnetic radiation to heat takes place by aligning the stochastic rotational dynamics of dipole moments (such as in water or DMF) and through ionic mobility within the synthesis environment [20,21]. The range of dipole moment frequencies (between 300 MHz and 300 GHz) causes the interaction between the microwaves and molecules [22]. All processes of absorbing electromagnetic energy and producing hotspots take place in less than a second with instantaneous uniform heat [23,24]. The uniform heat can be tuned by adjusting the electromagnetic radiation to oscillate the dipole moments of molecules [25].

**Figure 1:**
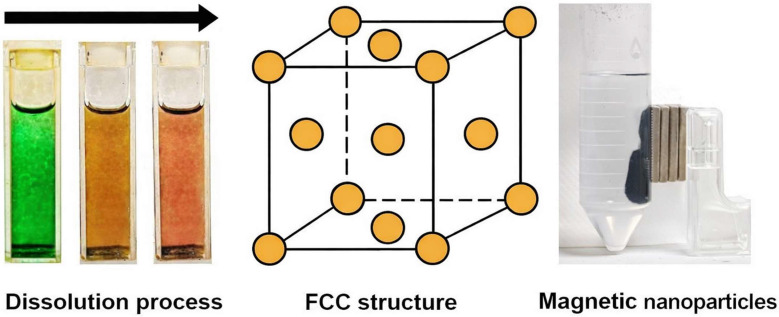
The dissolution process of [Fe_3_(CO)_12_] in DMF, which shows samples of the mixture at each stage, with the colour changes from dark green to wine red. The FCC structure corresponds to that observed in the magnetic Fe(0) nanoparticles (shown after washing to remove excess DDA, with collected nanoparticles and stirrer bar attracted to an external NdFeB magnet stack).

Here, we introduce a microwave-assisted rapid synthesis technique for producing Fe(0) nanoparticles from [Fe_3_(CO)_12_]. We leverage *N,N*-dimethylformamide (DMF) as an aprotic polar solvent to accelerate the dissolution of [Fe_3_(CO)_12_] and to conduct and propagate microwave radiation throughout the synthesis environment. Dodecylamine (DDA) was used during the production of the nanoparticles to coat and thereby stabilise them as metallic, magnetic Fe(0) nanoparticles with a face-centred cubic (FCC) structure with *Fm*$\bar{3}$*m* space group, as shown in Figure 1. Fourier-transform infrared (FT-IR) spectroscopy, powder X-ray diffraction (PXRD), annular dark-field transmission electron microscopy (ADF-STEM), and scanning electron microscopy (SEM) were used for analysis and characterisation.

## 2. Materials and Methods

### 2.1. Materials

Triiron dodecacarbonyl ([Fe_3_(CO)_12_], Merck, Glasgow, Scotland), anhydrous *N,N*-dimethylformamide (DMF, 99.8%, Scientific Laboratory Supplies Ltd., West Bridgford, UK), anhydrous acetone (Sigma-Aldrich, Gillingham, UK) ≥ 99.8%, max. 0.01% H_2_O), dodecylamine (98%, Sigma-Aldrich, Gillingham, UK), were used as received without further purification.

### 2.2. Instruments and Methods

A Monowave 200 (Anton Paar, Graz, Austria) high-performance microwave reactor with 850 W of power was used, along with a 30 mL (G30) reaction glass vial and a magnetic stirrer bar (500 rpm).

UV-vis spectra were recorded on a Perkin-Elmer Lambda (Waltham, MA, USA) spectrophotometer using a 2 nm slit width at a scan rate of 400 nm/min. The measurements were performed in 1 cm quartz cuvettes.

Attenuated total reflectance (ATR) and FT-IR spectroscopy (FT-IR) measurements were performed using a Bruker ALPHA platinum ATR spectrometer (Coventry, UK). FT-IR spectroscopy was performed at ambient temperature in a laboratory environment without N_2_ control. The sample vial, filled with N_2_, was quickly added to the FT-IR tip to minimise surface oxidation of Fe(0) and enable characterisation of surface functional groups, with air exposure estimated at less than 15 s (at ambient, no N_2_ purge).

PXRD data were collected on an Anton Paar XRDynamic 500 instrument (Graz, Austria) using Co Kα radiation (λ = 1.7889 Å) to avoid fluorescence from iron. Sample preparation and loading were carried out in an inert atmosphere (a glove box with an evacuated/back-filled antechamber). The powdered material was sealed in an N_2_-flow sample holder (chamber), and scans were recorded from 30 to 100 of 2θ/°. The PXRD analysis was conducted under N_2_ gas exposure at ambient temperature for the specified period. The profile fitting of the resulting diffraction patterns (Pawley refinement) was performed using a reference pattern (ICSD = 43,096) and the general structure analysis system II software (GSAS-II, version 5806, Python 3.13.3 64-bit, Argonne National Laboratory, Chicago, IL, USA) to assess the fit of the PXRD profile and the unit cell parameters [26].

SEM was carried out using a Zeiss Gemini (Karl Zeiss, Oberkochen, Germany) at a voltage of 5 kV with a resolution of 1 nm at 1 kV and 0.6 nm at 3 kV (auto carbon coater: EMITECH EVAPORATOR, Emitech Ltd., Ashford, UK) and Energy Dispersive X-ray Spectroscopy (EDX), which was acquired with 10 kV. A conductive silicon wafer (Agar Scientific, AGG3391, Rotherham, UK) was used to reduce the surface charge on Fe(0) nanoparticles. The samples were dispersed in 5 mL of anhydrous acetone and sonicated for 5 min at ambient temperature and pressure to disperse the metallic nanoparticles (500 W, Fisherbrand, FB15055, Leicestershire, UK). Then, a few drops of the sample were transferred onto a conductive silicon wafer in an inert atmosphere (a glove box with an evacuated/back-filled antechamber). The wafer was kept and sealed in an Eppendorf vial and quickly transferred to the SEM instrument, with an estimated exposure to air of 1 min during loading into the SEM.

STEM was used to determine particle size using a JEOL ARM200f double-aberration-corrected TEM (Tokyo, Japan), operated at 200 kV. Similarly to the SEM procedure, the sample was sonicated in 5 mL of anhydrous acetone, and a few drops were transferred onto TEM grids in a glove box, with an estimated exposure to air of 1 min during sample loading into the STEM.

Thermogravimetric analysis (TGA) was performed on a Mettler Toledo STARe (Leicester, UK) instrument from 273 K to 2773 K under air, with a flow rate of 50 mL/min.

### 2.3. Synthesis

All steps in the following synthesis were performed in a fume hood with HEPA filtration. [Fe_3_(CO)_12_] (150 mg, 0.298 mmol) was dissolved in 20 mL of anhydrous DMF in the 30 mL microwave glass vial and initially yielded a dark-green solution that rapidly turned brown and wine-red in colour within 1 min. Then, 550 mg (2.97 mmol) of DDA was added, and the 500 rpm stirrer bar was used to homogenise the reaction mixture (where the DDA did not fully dissolve at this stage). The vial’s vacant volume was purged with N_2_, then capped and sealed. The vial was transferred to a microwave reactor, and a 2.5 min ramp was used, with heating to 383 K, held for 30 s (with the highest power observed being 343 W), then cooled to 303 K (in the microwave reactor). No internal pressure was observed. After completing each synthesis, the sample was uncapped and briefly vented under N_2_ flow to release any evolved CO from the original [Fe_3_(CO)_12_]. The black precipitate, in the presence of excess DDA, can float in the vial. The sample was transferred to a 50 mL plastic centrifuge tube for excess DDA removal, performed by washing three times by centrifugation (6000× *g* rpm for 3 min) with anhydrous acetone (15 mL each time) and purging the evacuated volume in the centrifuge tube at each washing step with N_2_. The samples were dried in the centrifuge tube under a gentle flow of N_2_ at 292 K for 10 min. The black nanoparticle precipitate was recovered and demonstrated magnetic behaviour at ambient temperature (Figure 1).

The parameters for this synthesis, as described, were optimised for temperature and time. The introduction of a surface coating with DDA enabled the formation and stabilisation of Fe(0) but resulted in variable nanoparticle yield and final molecular weight.

## 3. Results and Discussion

High-purity Fe(0) nanoparticles were synthesised rapidly from [Fe_3_(CO)_12_] using a microwave reactor. Dissolution of [Fe_3_(CO)_12_] poses a significant challenge, as this precursor is notoriously insoluble in various solvents for this structure. By contrast, DMF can rapidly resolve the dissolution problem in [Fe_3_(CO)_12_] at ambient temperature within a minute. This new approach has been carried out using microwave-assisted synthesis at 368 K, with a hold time of only 30 s. This reaction was optimised for Fe(0) synthesis from 5 min to 30 s, and the size of the resulting nanoparticles and the volumes of the associated unit cells were analysed using electron microscopy and powder PXRD, respectively. Previous reports indicate that the synthesis of [Fe_3_(CO)_12_] is a time-consuming nucleation process, with sample preparation and synthesis taking a day [27]. Notably, the conventional method for Fe(0) nanoparticle formation needs several steps (from [Fe_3_(CO)_12_] to [HFe_3_(CO)_11_]) and is much slower, being 20 h at 473 K [27]. In addition, conventional methods need to be conducted in several steps, taking ca. 25 h of heating in the range 393–438 K, as reported by Amara et al. [28].

### 3.1. Dissolution of [Fe_3_(CO)_12_] in DMF

When anhydrous DMF was added to Fe_3_(CO)_12_ at ambient temperature (292 K), the colour of the solution changed from dark green to brown after 1 min, and then turned wine red and was transferred to a microwave reactor. Based on the study by Zacchini et al., dissolving [HFe_4_(CO)_12_]^3−^ in DMF results in a colour change to wine red, which yields the deep-red salt [Fe(DMF)_6_][Fe_4_(CO)_13_] [29]. DMF’s strong Lewis basicity displaces only the most labile carbonyls, the Fe(II) fragment paired with electron-deficient carbonyl anions, while allowing cluster growth [29,30].

**Figure 2:**
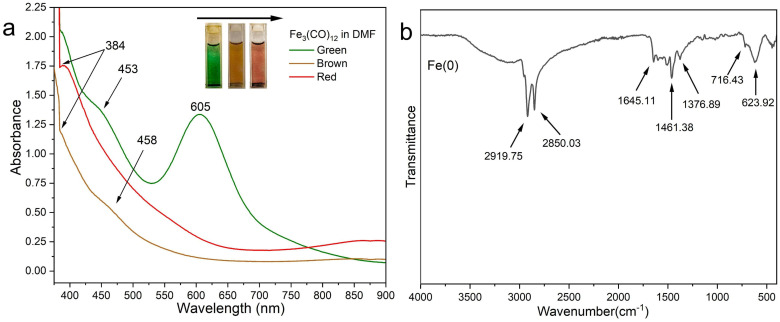
(**a**,**b**): (**a**) UV-vis absorption spectra of a DMF dissolution of [Fe_3_(CO)_12_] from dark green to red, and (**b**) the FT-IR spectrum of Fe(0) nanoparticles.

The UV-vis spectrum of [Fe_3_(CO)_12_] in DMF shows two absorption peaks at 453 nm and 607 nm, along with additional bands in the UV region, as shown in Figure 2a. As the colour changes during the dissolution process (green to red), the identical peak at 605 nm shifts and shrinks to 458 nm, accompanied by another band at 384 nm (in brown and red lines). These spectral changes suggest the formation of a DMF–Fe complex or cluster anion.

The shift of the 605 nm band to 545 nm matches the formation of the cluster anion due to the formation of [Fe(DMF)_n_]^−^ within a minute at ambient temperature. However, the colour changes in the [Fe_3_(CO)_12_] complex were reported by Watt et al. when it was treated with DDA in 1-octadecene. The solution changes from green to a wine red as [HFe_3_(CO)_11_]^−^ forms under a nitrogen flow and heating [27]. The study by Kim et al. shows that DMF coordinates with FeCl_3_ through the oxygen of carbonyl without reducing the Fe(III) in the solution [31]. FeCl_3_ in DMF remains as Fe(III) and forms a mixed chloro–DMF complex, and the isolated composition formulates as [FeCl_2_(DMF)_1.2_(H_2_O)_2.7_]^+^[FeCl_4_(DMF)_2.1_]^−^ [31]. These results show that DMF solvates (not reduces) Fe(III) and that multiple Fe(III) chloro–DMF ion-pair species coexist [31].

The Fe(0) does not exhibit any sharp, intrinsic bands in the FT-IR spectrum, as observed in Figure 2b. However, a band at 617 cm^−1^ in the fingerprint region is more consistent with surface Fe–O vibrations likely due to surface oxidation of Fe(0) nanoparticles, as reported in previous studies [32,33]. The small peak at 716.43 cm^−1^ is better assigned to the long-chain CH_2_ rocking mode from dodecylamine capping [34].

Multiple bands in the 1010–1034 cm^−1^ region are due to C–N stretching, while those between 1370 and 1500 cm^−1^ are attributed to CH_2_/CH_3_ bending (deformation). N–H (from the amine) appears as in-plane bending vibrations close to 1650–1580 cm^−1^ and out-of-plane wagging absorptions in the 909–666 cm^−1^ region [35,36]. The C–N stretching vibration of aliphatic primary amines appears between 1250 and 1020 cm^−1^ [36]. The peak at 1633 cm^−1^ is assigned to antisymmetric stretching modes of NH_2_, indicating that DDA is coated on the surface of the Fe(0) particles. In addition, the sharp bands between 2850 and 2920 cm^−1^ correspond to CH_2_ (alkyl chain), as reported by Ripmeester et al. [35]. A weak and broad absorption band is seen between 3100 and 3500 cm^−1^, corresponding to the N-H stretching vibration; however, this overlaps with the OH group. These bands can be assigned to DDA adsorbed on the nanoparticle surface during synthesis.

**Figure 3:**
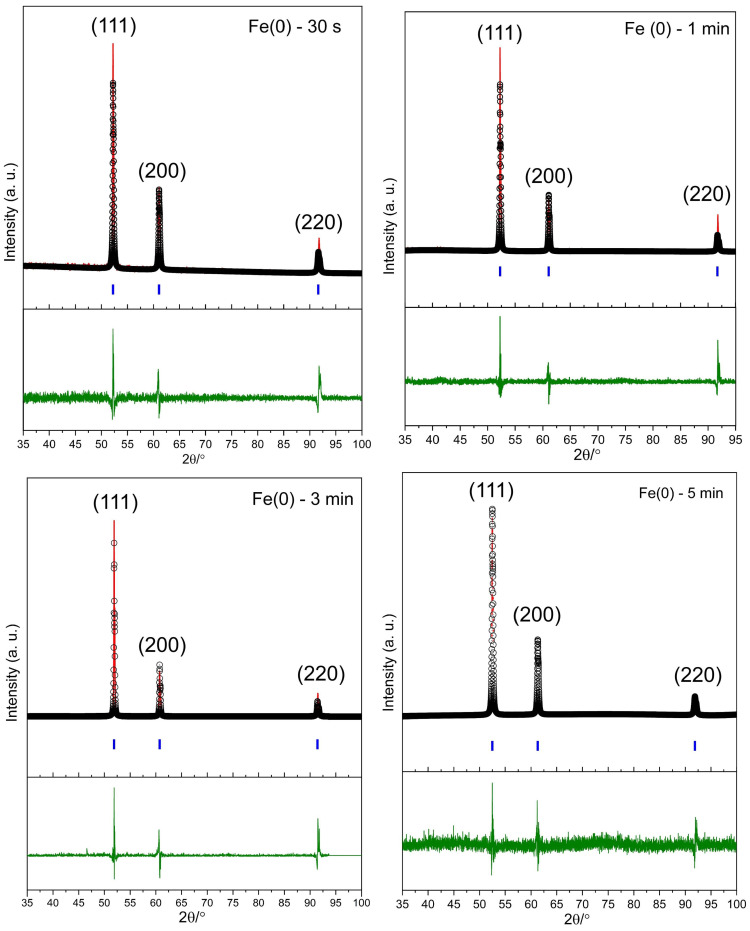
Fitted powder XRD patterns for the Fe(0) FCC structure with *Fm*$\bar{3}$*m* as the space group, as prepared at 383.15 K in 30 s, are presented, along with the experimental data (black points), Pawley refinement (red line), Bragg peak positions (blue), and a difference plot (green line).

The synthesis of Fe(0) nanocrystals using a microwave reactor and DDA as a stabiliser, resulting in the pure crystalline phase, is confirmed by PXRD. The unit cell parameters were obtained from a reference pattern (ICSD = 43,096), which is consistent with FCC iron, as shown in Figure 3.

As reported in previous publications, PXRD patterns show that using NaBH_4_ does not yield a pure Fe(0) phase and that it contains iron oxides as impurities [37,38,39,40,41,42,43]. Green synthesis routes, including green tea and Ricinus communis seeds, cannot produce pure Fe(0) crystalline phases [44,45]. Another synthesis approach that used [Fe_3_(CO)_12_] also indicated the presence of phase impurities, including oxide formation [28].

The PXRD patterns in four Fe(0) samples produced using different synthesis times, as illustrated in Figure 3, show three principal reflections at 2θ/° = 52.54 (111), 61.43 (200), and 92.09 (220) and an FCC structure with *Fm*$\bar{3}$*m* space group with a pure phase within the limits of detection. The peak positions of the samples produced with shorter (1 min and 30 s) synthesis are slightly shifted to higher 2θ values compared to the samples produced with longer (3 and 5 min) synthesis, which implies a small decrease in average d-spacings. The noisy background observed for all four samples is likely due to the DDA-coated layer on Fe(0) nanoparticles, as shown in Figure 2b.

Pawley refinements of the PXRD patterns indicate that the lattice parameter remains almost constant in all samples. The lattice parameters are 3.5276 (±0.0001) to 3.5391 (±0.0001) Å, and the volumes of unit cells are between 43.900 (±0.004) and 44.328 (±0.007) Å^3^; see Table 1. Based on previous studies, the volume of unit cells is between 46.063 and 49.77 Å^3^ for the FCC structure [46,47]. This suggests that rapid crystallisation and growth of Fe(0) nanoparticles in a microwave reactor lead to a smaller unit cell volume.

Due to the highly crystalline nature of the samples, the diffraction patterns were instrument-limited in width. GSAS-II could not define a meaningful crystallite domain size when peak widths were instrument-limited [26]. This is because sample size/strain broadening cannot be separated from the instrument profile [48]. This study shows that reducing the synthesis time allows for the retention of pure phases while maintaining the crystalline structure. Additionally, the use of DDA likely plays a role in stabilising the FCC structure in the Fe(0) crystals. Previous studies have shown that the use of DDA in Fe(0) synthesis can lead to an FCC structure [27,49]. Fe(0) normally adopts the BCC (α-Fe) structure at room temperature, and the FCC (γ-Fe) phase is stable in air only at high temperatures; however, there are a few reports that it can be stable at ambient temperature (1185–1667 K) [50,51].

### 3.2. SEM and STEM

**Figure 4:**
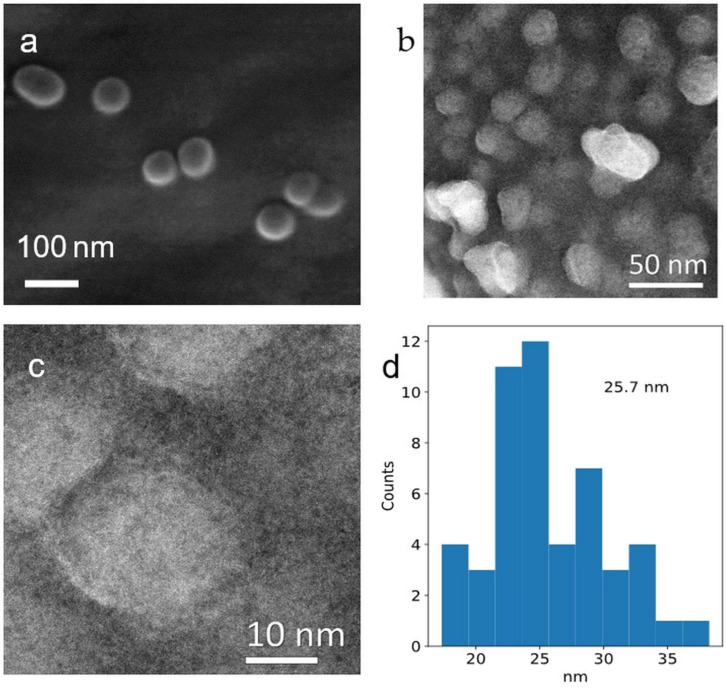
(**a**–**d**): Fe(0) nanoparticles synthesised in 30 s at 383 K, (**a**) SEM image with an oxide shell, and (**b**,**c**) STEM images (showing core–shell contrast from Fe core and DDA/oxide shell). (**d**) Particle size distribution histogram measured from STEM images, indicating an average size of 25.7 nm.

As shown in Figure 4a–d, the shape of Fe(0) nanoparticles appears homogeneous in both SEM and STEM images and the particle size histogram. The particles appear homogeneous and isotropic in both analyses; however, the images indicate some aggregation, likely due to the sample preparation process.

As shown in Figure 4a, a thin white shell around the particles is attributed to surface oxidation of Fe(0) nanoparticles and electron–beam interactions. This is relevant to the FT-IR results (Figure 2b), which show that the Fe-O peak appears at 617 cm^−1^, consistent with a thin oxidised shell. A study indicates that these oxygen-containing shell and beam-induced effects form a distinct pattern, influencing the final appearance of the particles in the micrographs [52].

The surface of Fe(0) nanoparticles in a STEM in Figure 4b,c shows that particle surfaces appear coated with a layer of DDA that covers Fe(0) nanoparticles. This layer caused interactions with the beam during analysis and also produced a white shell similar to those observed in the SEM image in Figure 4a. Based on the results of FT-IR spectroscopy (Figure 2b), the presence of an alkyl amine capping layer in 2920, 2850, and 720 cm^−1^ is assigned to asymmetric and symmetric CH_2_ stretching of long-chain alkanes. This coating helps produce non-flammable Fe(0) nanoparticles, as indicated during the synthesis.

A study on electron microscopy in the Fe-AC composite shows spherical Fe-core/carbon-shell nanoparticles with moderate agglomeration [53]. Additionally, a log-normal size distribution centred at 15 nm, and particle diameters of 6 nm, are present in the carbon matrix [53].

The mean particle size distribution is 25.7 nm, as measured from the STEM image shown in Figure 4d. A study by Lee et al. shows that the synthesised Fe(0) nanoparticles are highly aggregated, possibly due to the use of maximum power in that study (700 W) for a minute [54]. Similarly, ball milling (mechanochemical approach) can produce a pure PXRD phase; however, the size of particles is 100 μm with a high level of aggregation [9]. The use of NaBH4 in the synthesis can yield a heterogeneous phase, as observed by TEM, although the particle size is comparable to that in this study [43].

As shown in Figure S1, elemental analysis by EDX indicates that carbon, Fe(0), and oxygen comprise 53.3%, 15.3%, and 21.0%, respectively. This shows that the DDA adsorbed on the surface of Fe(0) nanoparticles, and the surface oxidation was lower than the amount of carbon. The TGA in Figure S2 shows that the weight loss starts at 99.5% at 273 K and ends at 2773 K with 46% weight loss, indicating that the DDA can stabilise the Fe(0) nanoparticles and coat their surfaces, as confirmed in Figure 4c.

Table 2 compares the results from our microwave-assisted synthesis method with those of similar previous studies. It highlights that it is possible to synthesise ultrafine nanoparticles with a pure, crystalline phase and to reduce the synthesis time, compared to other samples (FeCl_3_ and FeCl_3_·6H_2_O) that still require time for low crystallinity and phase purity.

However, the results from this table show that the metal ions can transfer microwave energy and produce homogeneous internal heat, unlike in conventional methods [56,57].

The rates of reaction in the microwave are estimated to be 1000 times stronger compared to conventional methods [58,59]. When polar media absorb microwave energy via dielectric loss, the molecules rotate rapidly, generate significant thermal effects, reduce activation energy, and weaken various chemical bonds [60].

## 4. Conclusions

This study reports that the choice of solvent and synthesis method can facilitate the development of a novel, rapid, simple process for producing Fe(0) nanoparticles. Use of DMF to dissolve [Fe_3_(CO)_12_] and form a new complex, fewer required stages to the protocol, and opportunities for venting of residual CO arguably provide safety advantages over alternative methods. This one-pot, low-energy approach provides uniform heating to accelerate the rapid formation of Fe(0) at lower temperatures than previously demonstrated, with phase purity within the limits of detection. The PXRD shows that the synthesised Fe(0) has the FCC structure with the *Fm*$\bar{3}$*m* space group. The Pawley refinement shows that the lattice parameters are almost constant between 30 s and 5 min, changing from 3.5276 (±0.0001) to 3.5391 (±0.0001) Å, and that the unit cell volumes range from 43.900 (0.004) to 44.328 (±0.007) Å^3^. The particle size predominantly consists of spherical particles between 10 and 50 nm, with an average of 25.7 nm, as observed via SEM and STEM. DDA plays a crucial role in stabilising the nanoparticles, providing a surface coating that prevents rapid oxidation (including flammability when exposed to air). This study can be extended to other metal carbonyl systems to produce metallic nanoparticles for various applications, as well as to continuous-flow synthesis for scale-up.

## Figures and Tables

**Table 1:** The Pawley refinement results from powder XRD data of Fe(0), the crystal domain size with FCC structure, with *Fm*$\bar{3}$*m* space group with varying the reaction time at constant heat (383 K, goodness of fit (GOF).

Time (min)	*a* (Å)	Unit Cell Volume (Å^3^)	GOF
5	3.5391 (±0.0001)	44.328 (±0.007)	1.55
3	3.5371 (±0.0002)	44.253 (±0.009)	1.56
1	3.5349 (±0.0001)	44.174 (±0.006)	1.75
0.5	3.5276 (±0.001)	43.900 (±0.004)	0.81

**Table 2:** Comparison of microwave-assisted Fe(0) synthesis with reported conventional methods.

Precursors	Solvent	Stabiliser/Reductant	Heat (K)	Time (s)	Power (W)	Ref.
FeCl_3_	Water	L. Mentha Piperita	321	180	N/A	[55]
FeCl_3_·6H_2_O	Water	NaBH_4_	N/A	60	750	[54]
Fe_3_(CO)_12_	DMF	DDA	368	30	243	This study

## Data Availability

The data presented in this study are available from the corresponding authors upon reasonable request.

## Supplementary Materials

The following supporting information can be downloaded at: https://www.mdpi.com/article/10.3390/nano16060353/s1, Figure S1: The EDX of analysis of Fe(0) nanoparticles on a conductive silicon wafer; Figure S2: The TGA of Fe(0) nanoparticles starts from 99.5% at 273 K and ends at 2773 K with 46% weigh loss.
