# Supplementary material for: Microwave-Assisted Rapid Synthesis of Metallic Iron Nanoparticles from Triiron Dodecacarbonyl
Source: Nanomaterials (Basel). 2026 Mar 13;16(6):353. doi: 10.3390/nano16060353 (PMC13028710; doi:10.3390/nano16060353)
Supplement: Supplementary file 1 [file nanomaterials-16-00353-s001.zip › nanomaterials-4151474-supplementary.pdf]

Microwave-Assisted Rapid Synthesis of Metallic Iron Nanoparticles from Triiron Dodecacarbonyl

Ehsan Ezzatpour Ghadim[a,b,]*, Yisong Han,[c] Festus Mathuen Slade[a,d,e]*

[a]*School of Engineering, University of Warwick, Coventry CV4 7AL, UK.*
[b]*Rapid Material Production Ltd, Coventry, CV1 3RZ, UK.*
[c] *Department of Physics, University of Warwick, Coventry, CV4 7AL, UK.*
[d]*Department of Chemistry, University of Warwick, Coventry, CV4 7AL, UK.*
[e]*Cambridge Neuroscience, University of Cambridge, Cambridge, CB2 1TN, UK.*

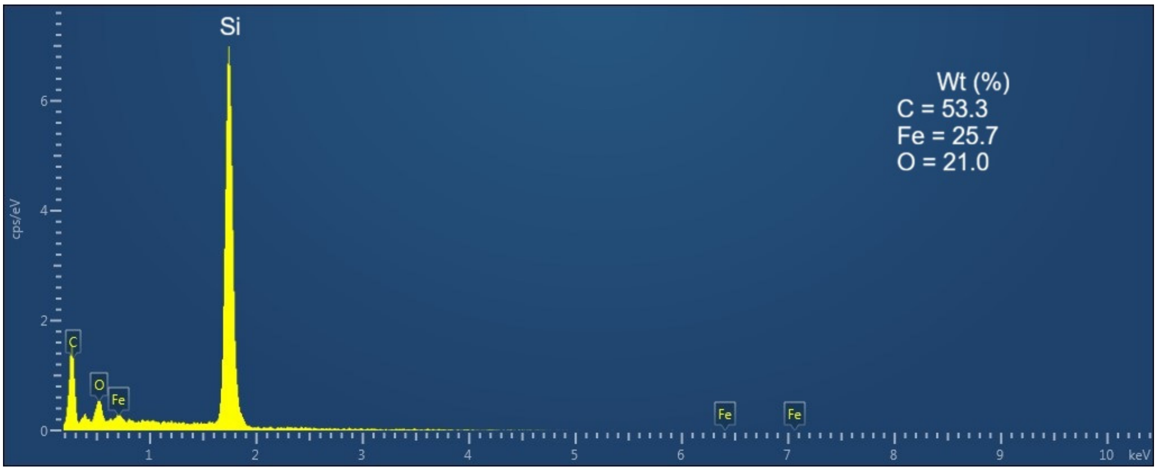

Figure S1: The EDX of analysis of Fe(0) nanoparticles on a conductive silicon wafer.

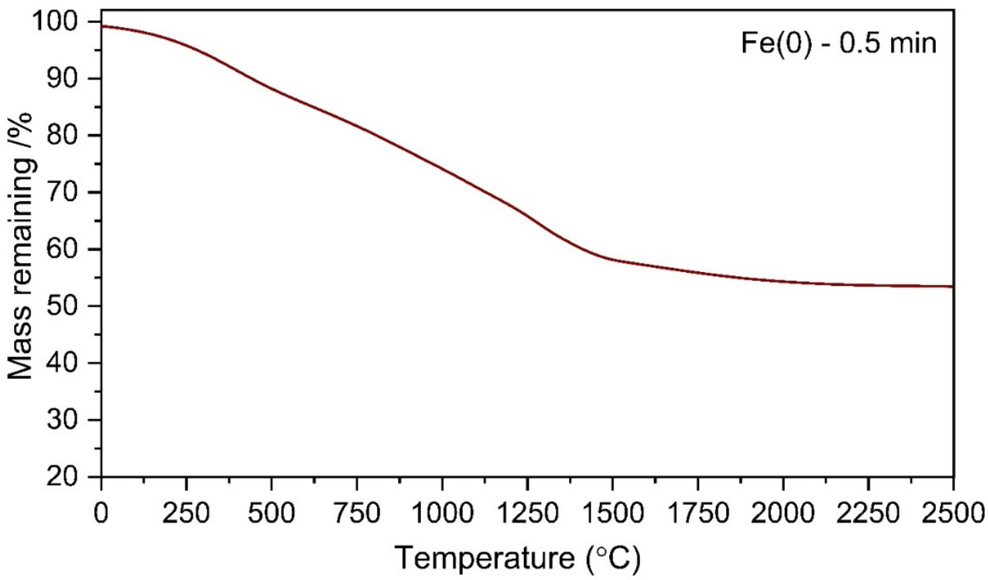

Figure S2: The TGA analysis of Fe(0) nanoparticles starts form 99.5% at 273 K and ends at 2773K with 46% weigh loss.
